# Versatile Dual Reporter Gene Systems for Investigating Stop Codon Readthrough in Plants

**DOI:** 10.1371/journal.pone.0007354

**Published:** 2009-10-09

**Authors:** Nga T. Lao, Alan P. Maloney, John F. Atkins, Tony A. Kavanagh

**Affiliations:** 1 Plant Molecular Genetics Laboratory, Smurfit Institute of Genetics, Trinity College, Dublin, Ireland; 2 Biosciences Institute, University College Cork, Cork, Ireland; 3 Department of Human Genetics, University of Utah, Salt Lake City, Utah, United States of America; University of Missouri-Kansas City, United States of America

## Abstract

**Background:**

Translation is most often terminated when a ribosome encounters the first in-frame stop codon (UAA, UAG or UGA) in an mRNA. However, many viruses (and some cellular mRNAs) contain “stop” codons that cause a proportion of ribosomes to terminate and others to incorporate an amino acid and continue to synthesize a “readthrough”, or C-terminally extended, protein. This dynamic redefinition of codon meaning is dependent on specific sequence context.

**Methodology:**

We describe two versatile dual reporter systems which facilitate investigation of stop codon readthrough *in vivo* in intact plants, and identification of the amino acid incorporated at the decoded stop codon. The first is based on the reporter enzymes NAN and GUS for which sensitive fluorogenic and histochemical substrates are available; the second on GST and GFP.

**Conclusions:**

We show that the NAN-GUS system can be used for direct *in planta* measurements of readthrough efficiency following transient expression of reporter constructs in leaves, and moreover, that the system is sufficiently sensitive to permit measurement of readthrough in stably transformed plants. We further show that the GST-GFP system can be used to affinity purify readthrough products for mass spectrometric analysis and provide the first definitive evidence that tyrosine alone is specified *in vivo* by a ‘leaky’ UAG codon, and tyrosine and tryptophan, respectively, at decoded UAA, and UGA codons in the *Tobacco mosaic virus* (TMV) readthrough context.

## Introduction

Termination of mRNA translation usually occurs when a stop codon (UAA, UAG or UGA) enters the A site of the ribosome. In eukaryotes, this process is mediated by two release factors: eRF1 which recognizes all three stop codons and triggers hydrolysis of the final peptidyl-tRNA bond, and eRF3 which stimulates eRF1 activity in a GTP-dependent manner [1]. Standard translational termination is a very accurate and tightly regulated process with an estimated failure rate of 10^−4^ [2]. However, its efficiency can be substantially altered by both *cis*- and *trans*-acting factors which include the nucleotide sequence context flanking the stop codon. While the identity of the 3′ adjacent base influences the effectiveness of termination [3], the identity of a more substantial context can be important for the synthesis of utilized readthrough products [4]. Examples of the more extensive context in viral decoding include such mRNA structural signals as a 3′ nearby pseudoknot [5], [6] and a structure comprising a 3′ distant sequence [7]. Factors that modulate GTPase activity [8], [9], [10], aminoglycoside antibiotics [11] and other factors [12] can increase the probability, by orders of magnitude, that a “stop” codon will be dynamically redefined. In readthrough the competition between decoding UGA, UAA and UAG by release factor and near-cognate tRNA, favours the latter.

In higher eukaryotes, perhaps the most remarkable examples of stop codon readthrough and its biological consequences are provided by the *Drosophila* developmental mutants *headcase* (*hdc*) [13], [14], *kelch* [15], and *out at first* (*oaf*) [16]. The corresponding wild-type genes encode two proteins translated from a single mRNA: a short termination product resulting from termination at the first in-frame “stop” codon and an extended readthrough form. For each of these genes, the efficiency of readthrough varies, in a tissue-, organ- or developmental-specific manner. The non-readthrough product (701kDa) of the *hdc* gene, for example, predominates in embryos, whereas in larval tracheal cells, high frequency UAA readthrough (20%) gives rise to a product (120 kDa) that functions as an essential inhibitor of tracheal branching. In the case of *kelch* and *oaf*, both of which are required for normal oogenesis, the efficiency of readthrough of a leaky UGA codon is also highly regulated. Thus, in testes and imaginal discs of 3^rd^ instar larvae, the kelch readthrough product (160 kDa) is almost as abundant as the non-readthrough protein (76 kDa), but only the latter is detected in ovaries, salivary glands and cuticle [15]; while for *oaf*, the highest levels of readthrough occur in larvae and pupae [16]. Comparison of *Drosophila* genome sequences has revealed an additional 149 readthrough candidates [17].

In plants, although candidate readthrough genes have been identified using computational approaches [18] and among gypsy-type retrotransposons from rice [19], all of the experimentally verified examples, to date, involve readthrough-mediated decoding of viral RNA genomes [20]. Members of the *Luteoviridae*, for example, synthesize short (22 kDa) and long (74 kDa) forms of the major viral coat protein, with the latter dependent on readthrough of a UAG codon. The non-readthrough product is sufficient for assembly of infectious virus particles, but the additional C-terminal domain generated by UAG redefinition is required for transmission of virions by aphids [21]. In contrast, members of the *Tobamoviridae* employ readthrough of a leaky stop codon in the replicase gene to regulate transcription and replication of the viral genome. Tobacco mosaic virus (TMV), the most intensively investigated example, synthesizes two replicase subunits: a 126 kDa non-readthrough protein (P126) containing methyltransferase and helicase domain, and a 183 kDa readthrough product, containing an additional C-terminal polymerase domain (P183) [22], [23]. A readthrough efficiency, *in vivo*, of approximately 10% ensures that P126 accumulates to a level approximately 10-fold higher than that of P183. This readthrough-dependent synthesis of P183, and maintenance of the P126∶P183 ratio, is critical for the viral life-cycle: mutants synthesizing only P126 are unable to replicate, while those synthesizing only P183 replicate poorly and are unstable [24], [25].

Various studies have shown that 25 nucleotides flanking the leaky stop codon are sufficient for readthrough of TMV RNA [26], [27] and identified the six consensus nucleotides CAR YYA located 3′ adjacent to the UAG codon as essential for readthrough [28], [29]. In addition, although the identity of the amino acid incorporated at the stop codon readthrough site *in vivo* in virus-infected plants has not so far been determined, several near-cognate tRNAs isolated from uninfected plants have been shown to decode stop codons in *in vitro* translation systems. A cytoplasmic tRNA^Tyr^ with a GΨA anticodon, purified from tobacco leaves, increased readthough translation of TMV *in vitro* in rabbit reticulocyte extracts from 10% to 35% [30], whereas no readthough was observed in wheat germ extracts which contain an abundant tRNA^Tyr^ with a QΨA anticodon. Two tRNA^Gln^ isolated from *Nicotiana rustica* were also shown to be active as TMV-UAG suppressors in wheat germ extracts [31], [32].

Important insights concerning the *cis*-acting sequence requirements and the mechanism of stop codon readthrough *in vivo* have been gained through the use of reporter gene constructs in which synthesis of the reporter protein depends on readthrough translation of an upstream leaky stop codon test sequence. For example, inserting oligonucleotides corresponding to viral readthrough regions into the 5′ end of the *GUS* reporter gene demonstrated that nucleotides located immediately 5′ and 3′ of the leaky stop codon act as critical determinants of readthrough efficiency [26], [28]. However, readthrough assays based on single reporter genes lack an internal control for initiating ribosomes and in consequence require careful monitoring of transfection efficiencies, reporter gene mRNA levels and translational efficiency between experiments. The use of a dual reporter gene strategy in which readthrough test sequences are placed between two reporter gene ORFs is therefore preferable because the upstream ORF acts as an internal control for translation initiation, simplifying between-experiment comparisons [27], [33], [34].

A dual reporter system suitable for investigating stop codon readthough in plants should ideally (i) provide sufficient sensitivity to detect readthrough at low levels following transient expression *in planta* (ii) facilitate histochemical detection of readthrough events in stably transformed plants; and (iii) yield sufficient quantities of affinity-purifiable readthough protein to facilitate mass spectrometry-based analyses. In this report we describe two dual reporter systems that between them meet the above criteria: the first is based on the reporter genes *NAN* and *GUS* [35], [36], for both of which fluorogenic and histochemical substrates are available; the second on the genes coding for GST and GFP [37], [38]. We show (a) that the NAN-GUS system enables facile measurement of the stop codon readthrough efficiency of candidate sequences *in planta* either following transient expression of test constructs in leaves, or in stably transformed plants; and (b) that the GST-GFP system can be used to affinity-purify readthrough proteins in sufficient quantity to enable mass spectrometry-based identification of the amino acid(s) specified by UAG, UAA and UGA.

## Results

### Investigating stop codon readthrough *in planta* using the *NAN* and *GUS* reporter genes

**Figure 1:**
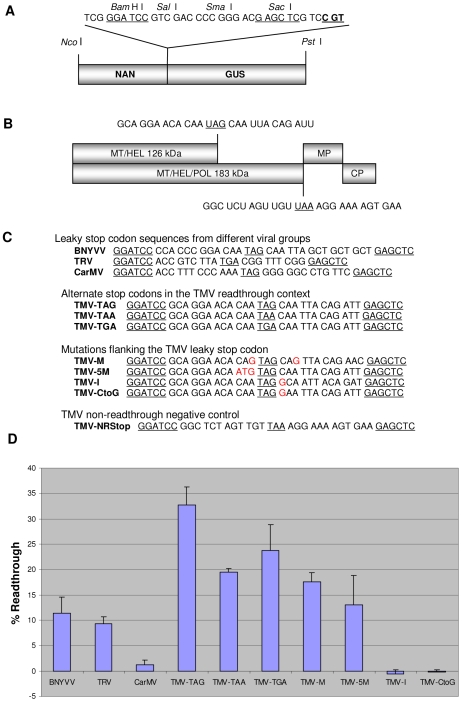
*In planta* measurement of stop codon readthrough using a NAN-GUS dual reporter vector. (A) Structure of the dual reporter gene cassette in the vector pOF. The *NAN* gene lacks a stop codon and a multiple cloning site separates the *NAN* and *GUS* orfs, which are translationally out-of-frame. CGT (bold and underlined) represents the 3^rd^ codon of the *GUS* gene. Test sequences were inserted between the *Bam*H I and *Sac* I restriction sites (underlined). (B) Diagrammatic representation of the TMV genome. The methyltransferase/helicase (MT/HEL) subunit of the viral RNA-dependent RNA polymerase is synthesized when translation terminates at a leaky UAG codon (underlined, with flanking sequence context). Stop codon readthrough extends the C-terminus of MT/HEL, adding the 57 kDa polymerase (POL) domain. Translation of the 183 kDa MT/HEL/POL protein terminates at a non-readthrough stop codon (NRStop) (underlined, with flanking sequence context). (C) Viral test sequences cloned as oligonucleotide linkers between the *NAN* and *GUS* orfs in pOF using the *Bam*H I and *Sac* I sites. Cloning sites and stop codons are underlined. Test sequences comprised leaky stop codon regions from the replicase orf of BNYVV, TRV and CarMV; the wild-type TMV readthrough region (TMV-TAG) and mutants with altered nucleotides (shown in red) at various positions flanking the leaky stop codon (TMV-5M, -I, -CtoG, -M); TMV mutants with alternate stop codons (TMV-TAA, TMV-TGA); and the region flanking the non-readthrough stop codon in the TMV replicase orf (TMV-NRStop). (D) Stop codon readthrough efficiencies of the various test sequences were calculated as described in the text. TMV-NRStop was used as a readthrough negative control.

We previously described the *NAN* gene, which codes for a highly active bacterial sialidase, as a sensitive activity-based reporter of gene expression in plants that can be used alone or in combination with the *GUS* gene and be assayed under the same conditions [35]. As a first step towards developing a dual reporter system to study translational recoding events, we investigated whether the NAN and GUS enzymatic activities are retained in NAN-GUS and GUS-NAN fusion proteins. Expression of the corresponding gene fusions in *E. coli* revealed that in both types of fusion protein the NAN and GUS specific activities were maintained at levels similar to that of the individual enzymes (data not shown). We therefore constructed a *NAN-GUS* expression cassette in which the reporter genes were separated by a multiple cloning site, and in which the downstream *GUS* gene was out-of-frame with respect to the *NAN* gene, so that the vector could be used to characterize translational recoding events such as stop codon readthrough and, in addition, frameshifting. The *NAN-GUS* out-of-frame cassette was cloned between the *Nco* I and *Pst* I sites of the provector module pICH11599 [39] to give pOF (Figure 1A).

### Stop codon readthrough efficiency of test sequences

Viral stop codon readthrough sequences were introduced between the *Bam*H I and *Sac* I sites of the pOF dual reporter vector (Figure 1A). The test sequences, derived from members of the virus groups identified previously [32] included: TMV and beet necrotic yellow vein virus (BNYVV), each of which has the consensus hexanucleotide CAA UYA at the 3′side of a leaky UAG codon (Type I); *Tobacco rattle virus* (TRV) which has CGG located 3′ of a leaky UGA codon (Type II); and *Carnation mottle virus* (CarMV) which has GGG GGC at the 3′ side of a leaky UAG codon (Type III). In addition to the wild type leaky sequences of the three above-mentioned types (i.e. constructs TMV-TAG, BNYVV, TRV, CarMV) we also tested a series of modified versions of the TMV readthrough region in which (i) the leaky UAG codon was replaced by UAA (construct TMV-TAA) or UGA (construct TMV-TGA), and (ii) critical nucleotide positions flanking the leaky stop codon were altered, as in the constructs TMV-CtoG, TMV-5M, TMV-M, and TMV-I (Figure 1C). A construct containing the non-readthrough stop codon (TMV-NRStop) sequence context that terminates translation of the TMV 183 kDa readthrough protein was used as a negative control (Figure 1B). Finally, for each of the above constructs, a corresponding positive (sense) control was generated in which the test sequence stop codon was replaced by CAG, which encodes glutamine (Q).

Readthrough efficiencies were calculated based on the GUS/NAN ratios of the test sequences, minus that of the TMV-NRStop negative control, normalized against that of the corresponding sense controls (Figure 1D). As expected, the GUS/NAN ratio of the NRStop negative control was found to be negligible, as was that of the pOF vector itself (data not shown). Readthrough efficiencies of 32%, 11% and 9% were observed for the wild-type TMV-TAG, BNYVV and TRV leaky sequences, respectively. Little or no readthrough was detected with the leaky sequence from CarMV. Replacement of the UAG in the TMV context with UAA or UGA reduced readthrough by at least 0.7-fold, whereas insertion of an additional base 3′ of the stop codon (TMV-I), or changing the C located immediately 3′ of the stop codon to a G (construct TMV-CtoG) abolished readthrough. In addition, changing the 5′ adjacent nucleotide and the third nucleotide 3′of the stop codon (construct TMV-M) reduced readthrough efficiency by more than 0.5-fold, confirming the important role played by the flanking CAA codons in determining readthrough efficiency [28]. Nevertheless, significant readthrough was observed for TMV-5M which was designed to rule out a simple tRNA hopping model that requires identical codons flanking the stop codon by replacing the glutamine codon (CAA) (putative take off site) on the 5′ side of the wild-type leaky stop codon with ATG.

### Detection of stop codon readthrough in stably transformed *Arabidopsis* plants

**Figure 2:**
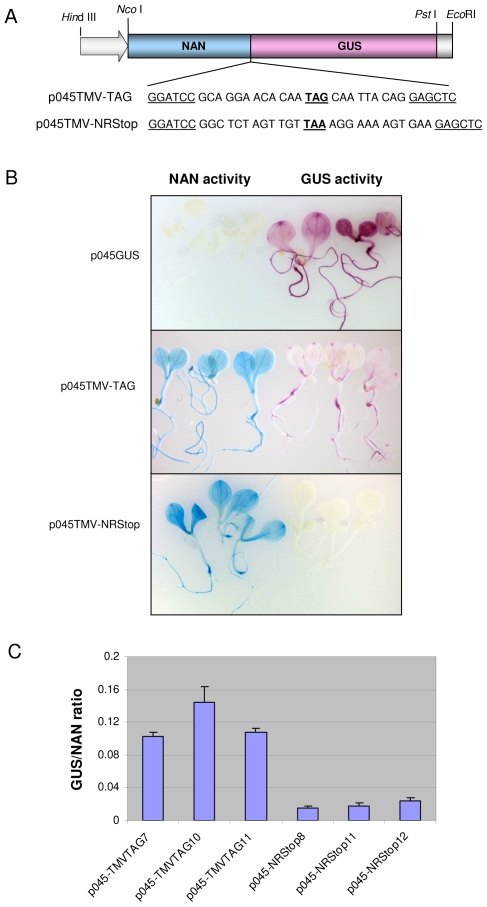
Detection of stop codon readthrough in stably transformed *Arabidopsis* plants. (A) Reporter gene constructs introduced into the *Arabidopsis* nuclear genome. The strong constitutive p045 promoter (open arrow) was used to drive expression of *NAN-GUS* dual reporter constructs containing either the wild-type TMV leaky stop codon region (p045:TMV-TAG) or the NRStop region (p045:TMV-NRStop) in stably transformed plants. (B) Histochemical analysis of progeny of single insertion transgenic *Arabidopsis* lines. Each panel contains six seedlings, 3 stained for NAN activity (left half) and 3 stained for GUS activity (right half). NAN activity stains blue, GUS activity magenta. Seedlings expressing a p045:GUS control construct show, as expected, strong GUS activity but no NAN activity (top panel). Seedlings expressing the p045:TMV-NRStop construct show strong NAN activity but negligible GUS activity (bottom panel), while seedlings expressing p045:TMV-TAG show strong NAN activity and low, but significant GUS activity (middle panel). (C) GUS/NAN activity ratios of seedlings of three individual transgenic lines expressing the p045:TMV-TAG (lines 7, 10, 11) or the p045:TMV-NRStop (lines 8, 11, 12) construct. NAN and GUS activity was quantified in total protein extracts from two pools of twelve seedlings of each line.

The high sensitivity with which readthrough could be observed via transient expression of NAN-GUS constructs, suggested it might also be detectable in stably transformed plants. This was investigated by generating constructs in which expression of the TMV-TAG and TMV-NRStop constructs was regulated by the strong constitutive p045 promoter from mung bean [40] (Figure 2A). A third construct containing only the *GUS* gene, regulated by the same promoter (p045GUS), was included as a GUS-positive, NAN-negative control. Constructs were introduced into the nuclear genome of *Arabidopsis* via *Agrobacterium*-mediated transformation and at least 12 independent transgenic lines per construct were analysed using NAN- and GUS-specific fluorometric and histochemical substrates.

For a given construct, all transgenic lines showed identical patterns of reporter gene expression as revealed by histochemical staining of seedlings and mature plants, but slightly different levels of total NAN and GUS activity. As expected, histochemical assays of lines containing the p045GUS construct showed strong GUS but no NAN activity or staining. The extent of stop codon readthrough was investigated by comparing the GUS/NAN activity ratios of progeny of three independent single-insertion transgenic lines containing the p045TMV-TAG construct with single-insert lines containing the p045NRStop construct. Histochemical staining revealed that p045TMV-NRStop lines showed the same pattern of NAN expression as lines containing the p045TMV-TAG construct, but no GUS staining (Figure 2B). Moreover, the GUS/NAN ratio of p045TMV-NRStop lines was less than 0.02, consistent with efficient termination of NAN translation (Figure 2C). However, histochemical staining of transgenic lines containing the p045TMV-TAG construct, expressing levels of NAN activity similar to that of matched p045TMV-NRStop lines, revealed low but easily detectable levels of GUS staining and a GUS/NAN activity ratio up to 6-fold higher than that observed in latter, consistent with a readthrough efficiency of 6–10% (Figure 2C). Although significantly lower than that observed in the transient expression system, this level of readthrough is comparable to that reported *in vivo* [25].

### Identification of the amino acid incorporated *in vivo* following mistranslation of UAG, UGA and UAA in the TMV readthrough context

**Figure 3:**
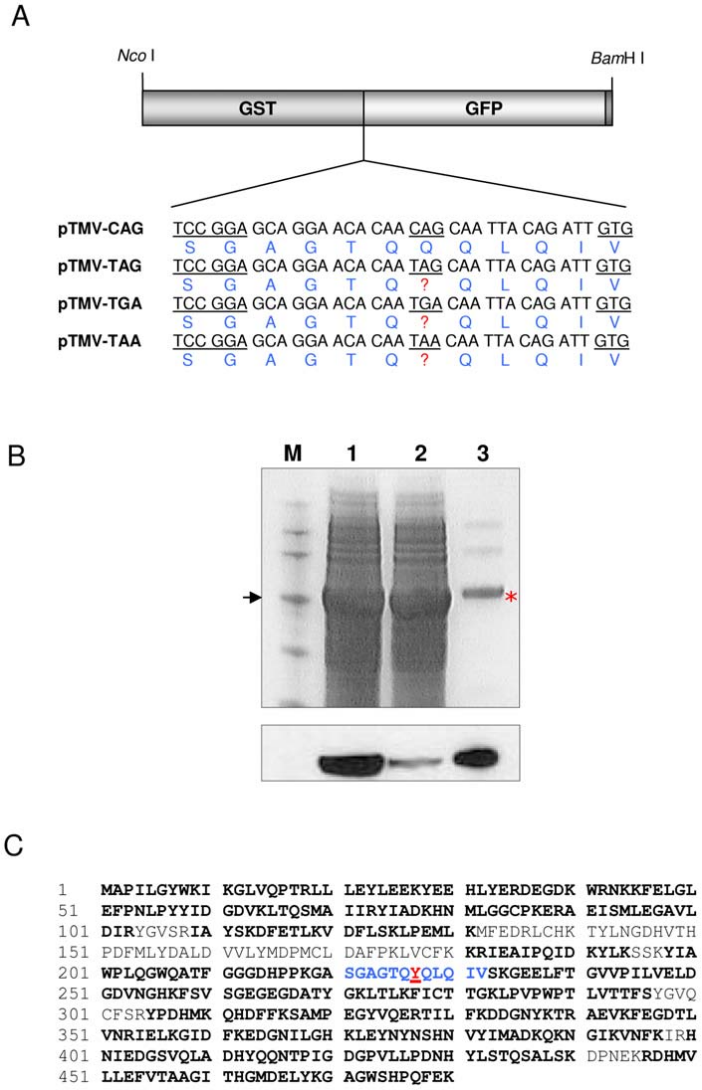
Identification of the amino acid inserted *in vivo* at a decoded stop codon using a GST-GFP dual reporter vector. (A) The GST-GFP dual reporter vector and the TMV test sequences incorporated between the reporter genes. Test sequences included the wild-type readthrough sequence (TMV-TAG), mutants containing the stop codons TGA (TMV-TGA) and TAA (TMV-TAA), and a sense control (TMV-CAG). Codons and the corresponding predicted amino acid sequences (blue) are shown; the *BspE*I cloning site and the second codon of GFP are underlined. The C-terminus of GFP is tagged with the StrepII-tag (shaded dark grey). (B) Analysis of GST-GFP readthrough proteins. Approximately 80 ug total soluble protein from *N. benthamiana* leaves harvested at 6 days post infiltration were separated on a 10% SDS polyacrylamide gel and stained with Coomassie blue (top panel). Readthrough proteins were detected by immunoblot analysis using HRP-conjugated Streptavidin (bottom panel). Lane M, Novagen™ Perfect protein marker (15–150 kDa); lane 1, TMV-CAG sense control; lane 2, TMV-TAG wild type leaky sequence; lane 3, StrepII tag-purified TMV- TAG readthrough protein (red asterisk). Arrow, 50 kDa marker protein. (C) Amino acid sequence of the purified TMV-TAG readthrough protein determined by in-gel tryptic digestion and LC/MS/MS analysis. Amino acids identified by LC/MS/MS are shown in bold; non-bolded letters indicate unidentified regions. The amino acid sequence corresponding to the test region inserted between GST and GFP is shown in blue. The amino acid tyrosine (Y) was identified at the leaky stop codon position (underlined, red).

Because of its large size (∼111 kDa), the isolation of sufficient quantities of the various NAN-GUS fusion proteins for LC-MS/MS-based analysis of the readthrough region proved to be problematic. We therefore employed an alternative dual reporter strategy based on the smaller reporter proteins gluthathione-S-transferase (GST) [37] and the jellyfish green fluorescent protein (GFP) [38], tagged at its C-terminus with the eight amino acid Strep-TagII epitope [41] (Figure 3A). Using this strategy, GST-GFP readthrough products (∼53 kDa) could be isolated easily by affinity capture using either glutathione-Sepharose or Strep-Tactin Sepharose.

Oligonucleotides corresponding to the TMV-TAG, TMV-TAA and TMV-TGA readthrough sequences and the TMV-CAG sense control were cloned between the GST and GFP orfs in the vector pGST-GFP (Figure 3A). Three week old *N. benthamiana* plants were infiltrated with *Agrobacterium* cultures carrying the various constructs and fusion proteins were purified from leaves harvested at 8 days post infiltration. The level of expression of the TMV-TAG readthrough product was approximately 20% compared with that of the sense control, estimated by SDS-PAGE and Western blot analysis (Figure 3B). We could easily obtain 1 µg affinity-purified readthough product from 1 g infiltrated leaves, although the level of accumulation of readthough products from the pTMV-TAA construct was slightly lower than that of the pTMV-TAG and pTMV-TGA constructs (data not shown). Purified readthough proteins were excised from Coomassie blue-stained polyacrylamide gels and subjected to in-gel tryptic digestion followed by mass-spectrometry analysis of peptides. Sequence coverage for the TMV-TAG readthrough product was 84% and tyrosine (Y) was identified as the amino acid incorporated at the leaky amber codon with a confidence of 6.2e-010 (Figure 3C). Similarly, sequence coverage for the TMV-TAA and TMV-TGA readthrough products was 77% and 88%, with tyrosine and tryptophan specified by the redefined codons respectively, with a similar level of confidence.

The possibility that stop codon readthrough in the TMV context might also occur by a “bypassing” (tRNA hopping) mechanism [28] was investigated by searching for peptides in which the leaky stop codon and the 5′ and 3′ flanking CAA codons were decoded by a single glutamyl tRNA; however, no such peptide was found. In addition, no peptide lacking an amino acid at a position corresponding to the flanking 5′ or 3′ CAA codons was detected.

## Discussion

To date, *in vivo* investigations of stop codon readthrough in plants have been based on measurements of GUS activity following delivery of readthrough-dependent single reporter gene constructs into protoplasts [7], [26]. Although yielding valuable information concerning the *cis*-acting sequences required for efficient readthrough, the use of protoplasts and the lack of an internal reporter control for initiating ribosomes limit the utility of this approach in several important respects. For example, it is not suitable for investigating readthrough in terminally differentiated tissues, or for investigating tissue- and/or developmentally-regulated differences in readthrough efficiency during the plant life-cycle. Furthermore, the very low-level expression of readthrough proteins in the protoplast system does not enable purification of quantities sufficient for downstream analyses. Here, we describe two novel dual reporter systems that address these limitations. Both systems are capable of yielding several micrograms of readthrough protein in less than a week, via transient expression in leaves, enabling visualisation of readthrough products directly, either by SDS-PAGE or via immunoblotting (i.e. without the need to employ *in vitro* translation).

The ease with which the NAN and GUS reporter activities can be quantified, their compatibility under the same assay conditions, and the availability of fluorogenic and histochemical substrates [35], make them ideal partners for measuring stop codon readthrough efficiencies either via transient expression or in stably transformed plants. All of the viral leaky stop codon sequences investigated, apart from CarMV, showed higher levels of readthrough than those reported using the GUS one-reporter system assayed in protoplasts [26]. Results for the TMV readthrough region were, however, comparable with those observed for the same region assayed in yeast using the dual reporter system based on β-galactosidase and luciferase [27]. Nevertheless, the fact that little or no readthrough was detected when the TMV leaky context was replaced with the corresponding non-readthrough region validates the suitability of the NAN-GUS system for *in vivo* assays of readthrough efficiency.

Synthesis of the TMV readthrough protein has been reported in various *in vitro* and *in vivo* systems including wheat germ extracts, rabbit reticulocyte lysates, tobacco protoplasts and *Xenopus* oocytes, despite significant differences in their tRNA complement [32]. As originally documented by Pelham [22], in the presence of purified amber (UAG) suppressor tRNAs from appropriate yeast strains, the rate of TMV readthough in the rabbit reticulocyte system increased from less than 10% to nearly 70%. In some of our experiments using the NAN-GUS system, readthrough of the TMV leaky region reached nearly 40%, and the readthrough product was visible on Coomassie blue-stained polyacrylamide gels (data not shown). We were also able to detect significant readthough of the TRV leaky region which was negligible in the GUS one-reporter system [26]. Moreover, the various TMV readthrough sequence mutations analysed gave readthough efficiencies similar to those reported previously [27], [28].

The GST-GFP dual reporter system was developed to facilitate affinity purification of readthrough products (∼53 kDa) that were less than half the molecular mass of the corresponding NAN-GUS readthrough proteins (∼111 kDa), and therefore more suitable for mass spectrometric identification of the amino acid incorporated at stop codon-suppressed sites. We identified tyrosine (Y) as the amino acid incorporated at both UAG and UAA, and tryptophan (W) at UGA in the TMV readthrough context, the first such direct identification in a plant system. In addition, we were able to investigate the possibility that readthrough translation of the TMV leaky region might be due to ‘stop hopping’ (rather than ‘stop decoding’), a process that would involve dissociation of the peptidyl-tRNA, slippage of the mRNA through the ribosome and re-engagement of the peptidyl-tRNA on a downstream matching codon [42]. Such an extended shift of the viral RNA could in principle take place during translation of the TMV readthrough region, since the slow-to-decode stop codon UAG is flanked by identical glutamine (CAA) codons that might provide compatible ‘take-off’ and ‘landing’ sites for a hopping peptidyl glutamine tRNA. However, the predicted hopping peptide product was not identified by mass spectrometric analysis of GST-GFP readthrough proteins, effectively ruling out a hopping mechanism and confirming previous results [28].

*In vitro* translation studies have shown that UAG or UAA in the TMV readthrough context can be decoded by several natural tRNAs from non-infected tobacco leaves including tRNA^Tyr^ (5′ GΨA 3′) [30], tRNA^Gln^ (5′ CUG 3′ or 5′ UmUG) [31], and also tRNA^Leu^ (5′CAA3′) from calf liver [43], implying apparent non-specificity of plant tRNAs for particular stop codons. The present study reveals that in the TMV readthrough context all three stop codons are efficiently decoded *in vivo*. However, while the natural tRNA^Gln^ has been shown to be the most potent tRNA decoder of the UAG stop codon in other systems, it apparently does not function in the readthrough of TMV-UAG *in vivo*, despite its presence in leaves, and despite previous studies showing readthrough *in vitro* by tRNA^Gln^ [31]. Instead, tRNA^Tyr^(GΨA) alone appears to act as a tRNA decoder of TMV-UAG and TMV-UAA via non-canonical base pairing at the first anticodon position [29]. Similarily, the reading of UGA by tRNA^Trp^(C_m_CA) depends upon a C_m_:A mismatch at the first position and no unconventional base interactions at the second or third anticodon positions [44]. This suggests that tRNA decoding of leaky stop codons in plant cells *in vivo* may be more stringent than in other eukaryotes.

In order to investigate stop codon readthrough in stably transformed plants, we used a strong constitutive promoter [40] to drive expression of NAN-GUS constructs containing the TMV leaky region in *Arabidopsis*. Readthrough expression of the *GUS* gene was easily detectable in the resulting transgenic lines using both fluorogenic and histochemical substrates. This is particularly interesting, as *Arabidopsis* lines expressing single transgene insertions of this construct could be used to identify genes involved in translational termination, or its regulation, by screening for mutants with altered GUS/NAN ratios. Recent studies in *Saccharomyces cerevisiae*, for example, have revealed mechanisms that modulate the synthesis of C-terminally extended proteins by regulating the efficiency of stop codon readthrough in response to cellular stress, a process that may impact on the ability of cells to respond and adapt to fluctuating environmental conditions (reviewed in [12]). Similar regulatory mechanisms may also play a role in enabling plants to adapt to environmental stresses.

## Materials and Methods

### Construction of the NAN-GUS dual reporter vector and introduction of test sequences

The *NAN* gene without its stop codon was amplified by PCR using the primers NANFNco (5′GCCATGGCTAACAAGAACAACACCTTCGAGAA; underlined is the modified second amino acid of the *NAN* gene in order to create a restriction Nco I site) and NANRBam (5′CGGATCCCGACATCTGCTTGTTAATAAGACTATAGT; underlined is the last codon of the *NAN* gene) and cloned into the expression vector pICH11599 at the unique *Nco* I and *Bam*H I sites. The resulting clone was named pICH11-NAN. The *GUS* gene without its start codon was amplified by PCR using the primers GUSOSac (5′ACGAGCTCGTCCGTCCTGTAGAAACCCCAA) and GUSRPst primers (5′TACTGCAGTCATTGTTTGCCTCCCTGCTGCGGT). The resulting PCR product was then used as a DNA template in a second round of PCR using the primers GUSOBam (5′CGGGATCCGTCGACCCCGGGACGAGCTCGT) and GUSRPst in order to engineer a multiple cloning site between the *NAN* and *GUS* orfs. The second PCR product was cloned into pICH11-NAN at unique *Bam*H I and *Pst* I sites and the resulting construct was named pOF.

Wild-type viral readthrough sequences and the corresponding sense controls were cloned into the dual reporter pOF vector as double-stranded oligonucleotides generated by annealing forward and reverse single-stranded pairs. Oligonucleotides were annealed at a concentration of 50 µM in 1 X TE at 90°C for 10 min and allowed to cool slowly to approximately 40°C before transferring to ice. Annealed oligonucleotides (1 pmole) were phosphorylated using T4 polynucleotide kinase in the presence of 1 mM ATP in 20 µl kinase buffer at 37°C for 30 min. Kinase activity was then heat killed at 65°C for 20 min. Each of the resulting double-stranded oligonucleotides contained a 5′ *Bam*H I and a 3′ *Sac* I single-stranded extension to facilitate cloning into the multiple cloning site of the pOF vector.

### Construction of the GST-GFP dual reporter vector and introduction of test sequences

The GST gene was PCR-amplified from pGEX-4T2 (GenBank accession U13854) with the forward primer GSTF (5′GCATCCATGGCTCCT ATACTAGGTTATTGGA), introducing an Nco I site (underlined) incorporating the translation initiation codon and the reverse primer GSTR (5′ATGCggatccTCATCCGGAAGCTCCTTTTGGAGGAT GGTCGCC) which introduced a *Bsp*E I site (underlined) immediately 5′, and a *Bam*H I site (lowercase) 3′, of the stop codon. The PCR product was digested with the restriction enzymes *Nco* I and *Bam*H 1 and the 670 bp fragment was cloned into the 3′ module cloning vector pICH11599, resulting in plasmid pICHGST. Four versions of the TMV recoding region incorporating a TAG, TGA or TAA stop codon, or the CAG codon specifying glutamine were cloned between the GST and GFP orfs, by amplifying an 800 bp GFP fragment using one of four different forward primers: TAG ( 5′GCTTCCGGA GCAGGAACACAA**TAG**CAA TTACAGATTGT GAGCAAGGGC ); TGA (5′GCTTCC GGAGCAGGAACA CAA**TGA**CAATT ACAGATTGTGAGCAA GGGC); TAA (5′GCATTCCGGAGCAGGAACACAA**TAA**CAATTACAGATTGTGAGCAAGGGC); or CAG (5′GCT TCCGGAGCAGGAACACAA**CAG**CAATTACAGATTGTGAGCA AGGGC) (*Bsp*E I sites are underlined) and the unique reverse primer GFPSTR (5′GATG CGGATCCTTATTTTTCAAATTGAGGATGAGACCAACCGGCGCCCTTGTACAGCTCG) which incorporated a *Bam*H I restriction site and the 8 amino acid Strep-tag at the GFP C-terminus. The resulting PCR products were cloned into pICHGST opened at *Bsp*E I and *Bam*H I generating pTMV-TAG, pTMV-TGA, pTMV-TAA and the positive control construct pTMV-CAG.

### Transient expression protocol

*N. benthamiana* seedlings grown for 3 weeks at 23 °C in a greenhouse with a 16-h/8-h light/dark cycle were used for transient expression of reporter gene constructs via agroinfiltration as described in [39]. Individual colonies of *Agrobacterium tumefaciens* strain GV3101 containing a reporter gene construct in pOF, the 5′ provector pICH10570 which supplies replicase functions, and the integrase-expressing vector pICH10881, were grown to OD_600_ 0.8 at 28°C in Luria-Bertani (LB) broth, containing 0.02 mM acetosyringone, 0.01 mM MES and 50 µg/ml rifampicin. Following centrifugation at 8,000 rpm for 10 min at 12°C, the cell pellet was resuspended in an equal volume of Infection Solution (IS) containing 0.1 mM acetosyringone, 0.01 mM MES, 0.01 mM MgCl_2_ and 50 µg/ml rifampicin. The cell suspensions were then incubated at room temperature for at least 4 hours. Prior to infiltration, equal volumes of the three provector cultures were mixed. Fully expanded leaves of 3 week-old *N. benthamiana* plants were co-infiltrated with the *A. tumefaciens* mixture using a 1 ml syringe without a needle. Plants were then grown under glasshouse conditions for up to 10 days. Each infiltration experiment was repeated 3 times.

### NAN-GUS readthrough constructs for stable expression in *Arabidopsis*

The 045 promoter region of the auxin-inducible aminocyclopropane-1-carboxylate (ACC) synthase gene of mung bean [40] was PCR-amplified from the plasmid p0.45GuNT using the primers P045HinF (5′CGCAAGCTTTACACGT GTAAAAAATA ATAGTTG ) and P045NcoR (5′GCCCATGGTAGGGACTGACCA CCCGGGGGAT) and cloned into the binary vector pFGC5491 (GenBank accession AY310901) at its unique *Hin*d III and *Nco* I sites, resulting pFGC-p045. The three constructs p045TMV-TAG, p045NR-Stop and p045GUS were generated by ligation of three restriction fragments: (i) the pFGC-p045 vector digested with *Nco* I and *Eco*R I, (ii) an *Nco* I-*Pst* I fragment containing the NAN-GUS genes separated by either the TMV-TAG or the NR-Stop sequence context; or the GUS gene alone and (iii) a *Pst* I-*Eco*R I nopaline synthase terminator fragment (amplified with the primer pair nosterF: 5′GCCTCGAGCT GCAGGAATTT CCCCGATCGTTCA and nosterR: 5′CGGAATTCCCGATCTAGTAA CATAGATGA CA (underlined letters indicate engineered restriction sites).

### *Arabidopsis* transformation

Stable transformation of *Arabidopsis* (ecotype Columbia) plants with *Agrobacterium* GV3101 containing NAN-GUS constructs was carried out using the floral dip method [45]. Transgenic lines were identified by spraying 1 week old seedling progeny grown at high density on soil with the selective herbicide BASTA (Hoechst, UK) at a final concentration of 100 µg/ml. Single insertion lines were identified by screening seedlings on MS agar medium containing 20 µg/ml DL-phosphinothricin (Duchefa, Holland).

### NAN and GUS activity assays

Total protein lysates from two pools of leaves (at 7 days postinfiltration) were prepared in lysis buffer containing 50 mM phosphate buffer pH 7, 10 mM β-mercaptoethanol, 10 mM disodium EDTA pH 8, 0.1% sodium laurylsarcosine, 0.1% Triton X-100, 1 mM PMSF). NAN and GUS activity assays were carried out in 96-well microtitre plates (FALCON 35 3075) in 100 µl assay buffer (50 mM phosphate buffer, pH 7; 10 mM β-mercaptoethanol) containing either 0.04 mM MUN or 0.1 mM MUG (Sigma, USA) as substrates, respectively, for NAN and GUS. Reactions were incubated at 37°C for 30 min, and terminated by adding an equal volume of 0.4 M Na_2_CO_3_. Methylumbelliferone (MU) fluorescence was measured (excitation at 355 nm, emission at 460 nm) using a FLUOstar OPTIMA fluorimeter (BMG Biotech, USA).

Histochemical visualization of NAN and GUS activity was performed using the substrate 5-bromo-4-chloro-3-indolyl-α -D-N-acetylneuraminic acid (X-NeuNAc) (Sigma, USA) for NAN and 5-bromo-4-chloro-3-indolyl-β-D-glucuronide (X-GlucA) (Duchefa) or 5-bromo-6-chloro-3-indolyl-β-D-glucuronide (X-GlucM) (Glycosynth, UK) for GUS as described previously [35].

### Purification and mass spectrometry analysis of Strep-tagged GST-GFP readthrough proteins

Infiltrated leaves were harvested at 8 dpi, frozen in liquid nitrogen and ground to a powder. The powdered material was further homogenized in Strep Buffer (SB) which contained 100 mM Tris-HCl (pH 8.0), 150 mM NaCl, 10 mM EDTA, 10 mM β-mercaptoethanol, 1 mM PMSF at a ratio of 3 ml per g of leaf material. Strep-tagged proteins were identified following SDS-PAGE by Western blot analysis using a horseradish peroxidase-conjugated primary anti-StrepIITag antibody (IBA, Germany). Strep-tagged readthrough proteins were purified by affinity chromatography as follows: cell extracts were clarified by centrifugation at 12, 000 x *g* for 10 min at 4°C. The supernatant was filtered sequentially through Miracloth, 0.8 and 0.2 µm filters prior to loading onto a Strep-Tactin Sepharose column (IBA, Germany). Columns were equilibrated with SB before cleared protein lysates were applied. Bound Strep-tagged proteins were eluted with Strep Elution Buffer (100 mM Tris-HCl,150 mM NaCl, pH 8 and 2.5 mM desthiobiotin). Eluted proteins were desalted and concentrated using Macrosep 10K Omega centrifugal devices (Pall Life Sciences, USA). Concentrated affinity-purified protein samples (approximately 2 µg) were fractionated by SDS-PAGE, the proteins visualized by Coomasie blue staining, and the relevant bands excised for in-gel trypsin digestion and LC/MS/MS. This analysis was carried out at the University of Utah using a LTQ-FT mass spectrometer. Detailed analysis and identification of peptides was performed using MASCOT software.
